# Is Ortho-Terphenyl a Rigid Glass Former?

**DOI:** 10.1021/acs.jpclett.4c01217

**Published:** 2024-07-01

**Authors:** Johanna Kölbel, Michael T. Ruggiero, Shachar Keren, Nimrod Benshalom, Omer Yaffe, J. Axel Zeitler, Daniel M. Mittleman

**Affiliations:** † School of Engineering, Brown University, Providence, Rhode Island 02912, United States; ‡ Department of Chemistry, University of Rochester, Rochester, New York, 14627, United States; § Department of Chemical and Biological Physics, Weizmann Institute of Science, Rehovot 7610001, Israel; ∥ Department of Chemical Engineering and Biotechnology, University of Cambridge, Cambridge CB3 0AS, U.K.

## Abstract

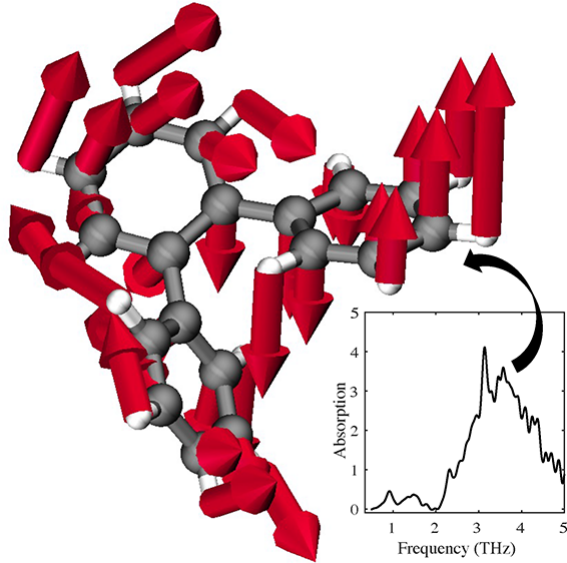

Ortho-terphenyl (OTP) has long been used as a model system to study the glass transition due to its apparent simplicity and a widespread assumption that it is a rigid molecule. Here, we employ terahertz time-domain spectroscopy and low-frequency Raman spectroscopy to investigate the rigidity of OTP by direct observation of the low-frequency vibrational dynamics. These terahertz phonons involve complex large-amplitude atomic motions where intramolecular and intermolecular displacements are often mixed. Comparison of experimental results with density functional theory and *ab initio* molecular dynamics simulations shows that the assumption of rigidity neglects important implications for the glass transition and must be revisited. These results highlight the significance of terahertz modes on elasticity, which will be even more critical in more complex systems such as biomolecules.

Understanding the formation of a glass from a liquid melt is one of the most challenging problems in materials physics. As a molecular liquid is cooled through its glass transition, both the mobility and internal degrees of freedom of the molecules change dramatically, despite the fact that there is little change in overall structural arrangement at a molecular level. In order to simplify the problem, one approach is to minimize the role of those internal degrees of freedom, to eliminate complexities such as vibrational mixing^1^ between internal and external modes or anharmonic mode coupling.^2^ Unfortunately, the simplest systems, atomic liquids, crystallize at low temperature. As a result, many researchers have sought to study simple molecular systems which are as rigid and spherical as possible,^3^ for comparisons with theoretical models in which such couplings are neglected or considered only as perturbations. Among these, mode coupling theory (MCT) has become a popular framework.^4^ MCT suggests that damping of the density correlations is mediated by a nonlinear coupling, which results in an extreme sensitivity of the relaxation dynamics to small changes in structure and temperature. However, there is still considerable debate about the role of internal molecular degrees of freedom. This calls into question some proposed mechanisms in cases where the glass-forming molecule is not as rigid as previously assumed. One widely studied glass-former is ortho-terphenyl (OTP, C_18_H_14_).^5−7^ It consists of two phenyl groups that are twisted in the same direction with respect to a central phenyl group.^8^ Its melting point (approximately 329 K^9^) and glass transition temperature (243 K^10,11^) are easily accessible experimentally. Its atomic structure is relatively simple, and benzene rings, in particular, are usually very stable.

In 1994, the so-called Lewis-Wahnström ortho-terphenyl model therefore proposed that the OTP molecule can be represented by a three-site complex, each site playing the role of a whole benzene ring. This model neglects internal degrees of freedom, i.e., the rigid-molecule approximation is invoked, in order to simplify calculations.^12^

Following this, a large number of studies have relied on the same assumption of rigidity; indeed, OTP has become a model system for the study of the glass transition.^13,14^ For instance, the rigid approximation of the Lewis-Wahnström model for OTP was used in molecular dynamics simulations of rotational dynamics,^12^ to investigate translational and rotational diffusion,^14^ and to characterize crystallization.^15^ Experimental studies have also employed this framework, such as in measurements of specific heat and thermal conductivity^16^ and studies of translational and rotational diffusion in supercooled OTP.^6^

These minimally complex models were able to provide physical insights and reproduce some of the complexity of real systems with minimal computational cost.

However, controversy remains about the underlying assumption of rigidity of OTP. For example, a neutron scattering study on deuterated OTP^6,17^ concluded that the intramolecular phenyl ring motion is not the dominant mechanism for the glass transition β-process, thus supporting the idea of center-of-mass motion as the key parameter of the glass transition.^6^ On the other hand, a NMR study on deuterated OTP found that the end rings can undergo “flips” even in the crystalline phase,^18^ which points to the importance of intramolecular motions. OTP is still being used as a model system, for example, for evaluating coarse-grained models,^19^ studying surface mobility,^20^ or investigating statistical mechanical theories of the glass transition.^21^

It has been suggested that the potential energy surface (PES) plays an important role in dictating the atomic dynamics and structures in glassy materials.^22−25^ A shared fundamental origin, such as the PES, can explain why glass transition temperatures can be measured with a variety of techniques probing completely different time and length scales, for example, neutron scattering,^26^ Brillouin light scattering,^27^ dynamical mechanical analysis,^28^ Raman scattering,^29^ and THz-TDS.^30^ In organic molecular solids like OTP, low-frequency mode types are often a mixture of intra- and intermolecular dynamics.^31^ The shape and structure of the PES, both intra- and intermolecular, therefore defines the complete intermolecular dynamics.

Glass transition temperatures are fundamental characteristics of the PES, as are (terahertz) modes and the observed elasticity. *T*_g,β_ has been connected to localized structural changes corresponding to sufficient energy to overcome small potential-energy barriers, and *T*_g,α_ has been connected to large-scale conformational rearrangements and changes between different “basins” on the PES.^32^ By measuring terahertz modes, we sample the PES and thereby the same coordinates that underpin elasticity. The question is therefore which (terahertz) vibrational potentials and dynamics are important for understanding glass and phase transitions.

This does not only apply to OTP, which we are using as a model system. Indeed, understanding glass and phase transitions (and the role that elasticity plays in those) is a fundamental question of interest, and low-frequency vibrational spectroscopies such as low-frequency Raman and THz-TDS are promising tools.

Terahertz spectroscopy has been previously employed, for example, to study the elasticity of human corneas,^33^ characterize the flexibility of MOFs^34^ (which has implications, for example, for gas storage and separation applications), or to understand the stability of protein secondary structures.^35^ Protein structural stability, mechanical strength, and catalytic activity have all been linked to elasticity.^36−41^

Here, we describe a combined experimental and computational study of the internal degrees of freedom in OTP and their role in glass formation. We utilize a suite of techniques to investigate this, notably both low-frequency Raman and infrared spectroscopy in the terahertz range (1 cm^–1^ to 150 cm^–1^).

This is significant because torsional motions (in the form of internal movement of OTP’s phenyl rings) fall in this spectral range. As a consequence of the mixing intra- and intermolecular displacements, the deconvolution of individual atomic contributions in experimental spectra is not possible without additional information gathered from theoretical simulations.^42^ The combination of low-frequency spectroscopies with computational methods such as density functional theory (DFT) and *ab initio* molecular dynamics (AIMD) simulations provides a powerful joint approach to uncover the complete picture of low-frequency dynamics in crystalline solids.^42,43^

Here, we show that low-frequency vibrational spectroscopy is a promising choice for investigating the role of intramolecular degrees of freedom in glass-forming systems. We show that terahertz modes do not only play a large role in macromolecules, but already influence the model system OTP, highlighting the importance of internal and external modes and their impact on elasticity in even simple systems.

*THz and Raman spectroscopy of crystalline OTP*. We obtain spectra of pure crystalline OTP (space group *P*2_1_2_1_2_1_, 4 molecules per unit cell, used as purchased) in the 0.1–5.0 THz range using a conventional time-domain spectrometer (TDS). In these spectra, acquired at 80 K, distinct spectral features are visible at 0.9 THz, 1.5 THz, 2.3 THz, 3.1 THz, and 3.5 THz, as shown in Figure 1a. At 300 K (room temperature), the features at 0.9 THz and 2.3 THz are shifted and broadened, as apparent in Figure S1.

**Figure 1:**
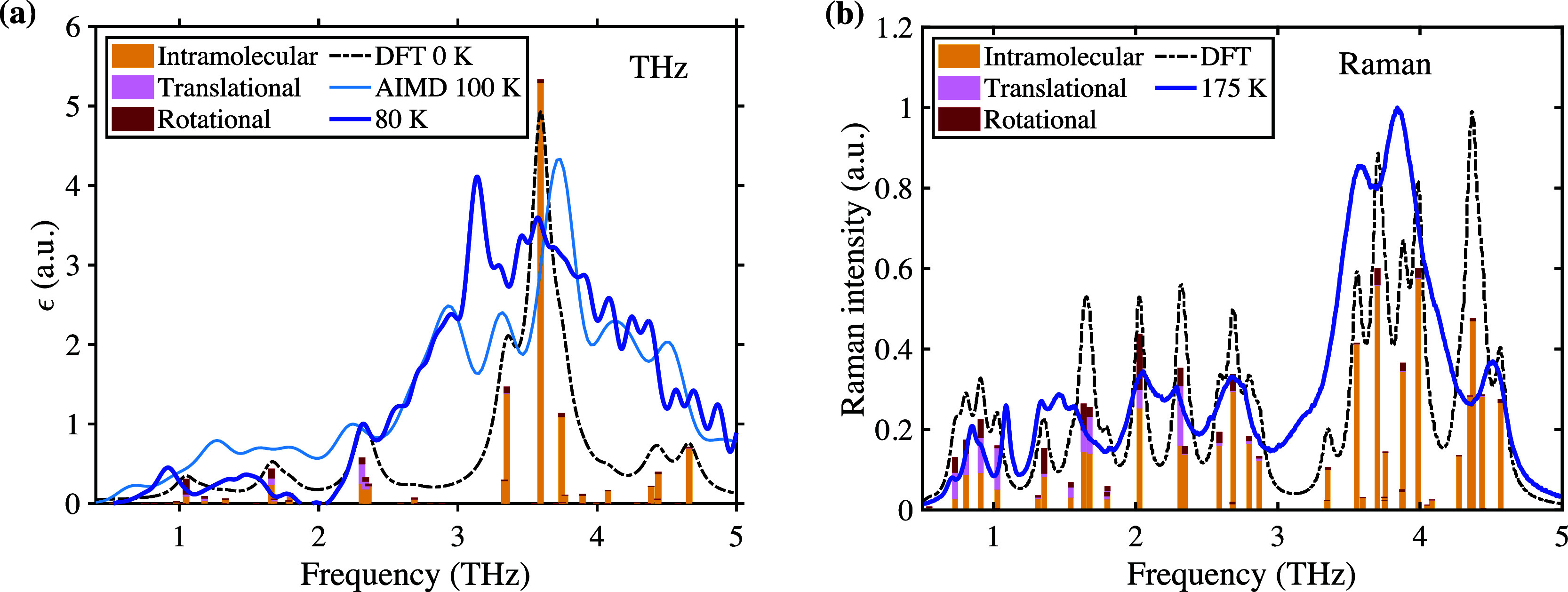
(a): Measured (dark blue) and calculated (light blue, black) terahertz spectra. Relative contribution of inter- and intramolecular modes as calculated from DFT are shown as sticks. All intensities have been scaled to the feature at 2.3 THz. (b): Raman intensity measured at 175 K (blue), and calculated with DFT (black). Sticks show relative contributions of inter- and intramolecular motions.

Because mode assignments based on simulations will give insights into the relationship between specific terahertz modes and the rigidity of OTP, we calculated vibrational intensities by DFT utilizing the CRYSTAL23 software package with the Perdew–Burke–Ernzerhof (PBE) density functional,^45^ Ahlrich-VTZP basis set,^46^ D-3 dispersion correction,^47,48^ and periodic boundary conditions,^49^ as described in more detail in the Supporting Information (see also refs^8,45−55^ therein). To facilitate comparison of the simulated and experimental results, the intensities were convolved with a Lorentzian whose fwhm equals the bandwidth of the instrument (see Figure 1a).

These DFT calculations are performed within the harmonic approximation and do not incorporate thermal effects. We therefore use AIMD simulations to study the effect of temperature on the inter- and intramolecular mobility and elasticity of OTP. Figure 1a also includes the spectrum calculated from AIMD simulations performed at 100 K utilizing the CP2K software package,^56,57^ the PBE functional,^45^ DZVP basis set,^58^ and Goedecker-Teter-Hutter (GTH) pseudopotentials for core electrons,^59^ as described in more detail in the Supporting Information (see also refs^45,48,56−61^ therein). The feature at 2.3 THz is reproduced very well by both simulations, and the overall agreement with the experiment is good, suggesting that a more in-depth analysis of the simulated results is warranted, to illuminate the impact of internal modes on elasticity in OTP.

As a complementary technique to THz-TDS, low-frequency Raman spectroscopy probes modes involving a change of polarizability. We have measured the Raman shifts in a home-built system using backscattering measurement geometry, with a 785 nm CW diode laser (Toptica Inc., USA). The spectrum at 175 K is shown in Figure 1b (scaled to the most intense feature at around 3.9 THz). We also observed some peak narrowing and shifting compared to room temperature (see Figure S2). The experimental spectrum is again reproduced well by the DFT simulation, although the relative intensities at lower frequencies are slightly underestimated. We note that simulations at terahertz frequencies require very tight convergence criteria as well as dispersion correction.^62−65^ The agreement between simulations and measurement once again indicates the quality of the computational results, which we use to examine the mode character and elasticity in more detail.

Once the vibrational eigenvectors of a molecular crystal are determined from DFT simulations, it is possible to determine the fractional contribution of inter- and intramolecular motions to the normal modes, and further to separately quantify the translational and rotational/librational contributions to the intermolecular motion.^66^ We have performed this analysis for each of the modes of OTP, with results shown as histograms in Figure 1. Below 3 THz, the observed IR-active modes consist of approximately 50% intramolecular character, whereas above 3 THz, intramolecular motion contributes to more than 90% of each mode. The Raman-active modes are also highly intramolecular, and a mix of inter- and intramolecular modes dominates at frequencies below 2.5 THz. A frequency of four THz corresponds to a vibrational temperature of 192 K, therefore internal modes at frequencies below 4 THz are all thermally excited at room temperature (occupied to at least 50 %) and therefore contribute to molecular mobility and elasticity. The glass transition temperature of OTP lies below room temperature (243 K^10,11^). Correspondingly, internal and external modes are active in the disordered state as well as in the energetically lower crystalline state. The AIMD simulations of the terahertz spectra at different temperatures confirm this by showing an increase in the background absorption (sometimes referred to as vibrational density of states), just as observed experimentally, and usually attributed to disorder-induced coupling of the terahertz radiation to Debye-like acoustic vibrational modes.^67,68^ They also show some peak shifting above 2 THz with an increase in temperature (Figure S3).

AIMD simulations inherently account for anharmonicity, but lack mode-specific information.^43,63,69−71^ We used the AIMD simulations as the basis to calculate a vibrational spectrum at different temperatures.^60,61^ Methods for projecting normal modes from molecular dynamics simulations are not currently available in any existing DFT package, as far as the authors are aware. While extracting changes in dihedral angles from the AIMD trajectory is possible, we cannot easily assign these movements to single modes.

DFT simulations, on the other hand, directly provide normal modes. These allow us to investigate mode-specific torsional motions, such as changes in the dihedral angle between the side and central rings. Those are explicitly excluded in the Lewis-Wahnström model. We note that the AIMD and DFT simulations predict features which are situated very closely to those found in the experimental spectrum (see, e.g., the IR-active modes at 2.3 THz, or the high-intensity modes around 3.5 THz). We did not have to apply a scaling factor to the simulated frequencies to observe this agreement and are hence confident that the simulated modes are very similar between the two methods we utilized. The AIMD simulations at 300 K show an especially increased absorption at frequencies above 3 THz. As can be seen in Figure 2, modes at these frequencies particularly correlate with internal torsions. These also occur at lower frequencies and hence already contribute to the internal modes in OTP at very low temperatures.

**Figure 2:**
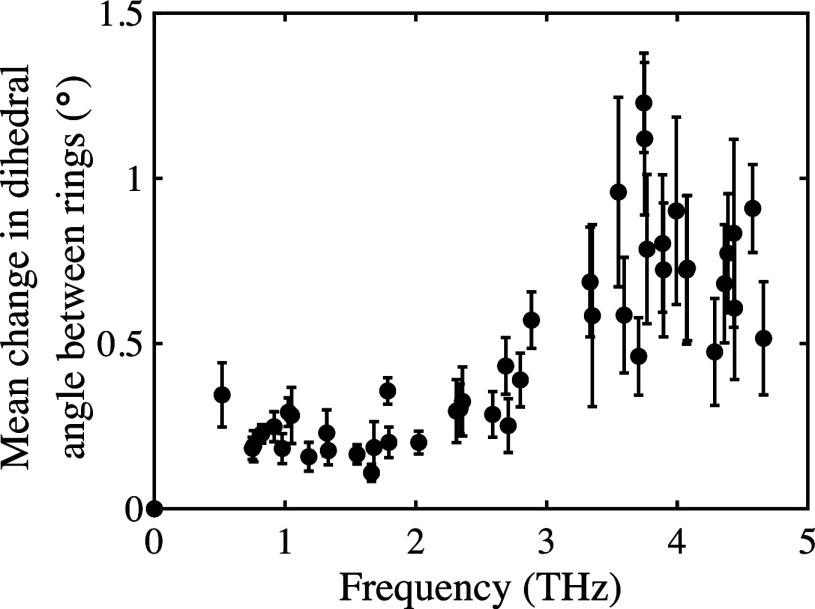
Average change in dihedral angles between side and central rings for IR and Raman active modes, extracted from DFT simulations for one unit of displacement. Error bars are standard error of the six dihedral angles studied. A larger error bar indicates that not all dihedrals are changing equally.

*Young’s modulus and elasticity tensor*. In addition to the spectroscopic studies noted above, we also performed a nanoindentation measurement perpendicular to the [200] facet of a single crystal of OTP grown from methanol solution according to the procedure outlined in ref (72). We extract from these measurements a Young’s modulus of 13.8 GPa ± 1.0 GPa. We compare this with a calculated result extracted from the DFT simulations, by determining the elasticity tensor with CRYSTAL23 (keyword ELASTCON) which allows the computation of Young’s modulus for different strain directions relative to the crystal axes.^73^ The calculated value of 13.8 GPa again validates the accuracy of our theoretical methodology. The anisotropy of Young’s modulus for arbitrary strain directions is visualized in Figure S4, which shows that the crystallographic directions of maximum and minimum stiffness are perpendicular to the [100] and [001] facets, respectively.

Next, we calculated the direction of the center of mass (COM) movement of the entire molecule as well as of each of the three rings individually. We used these results to compute the Young’s modulus in the respective direction. These predicted values are shown in Figure 3 for selected modes. Details about the calculations can be found in the Supporting Information.

**Figure 3:**
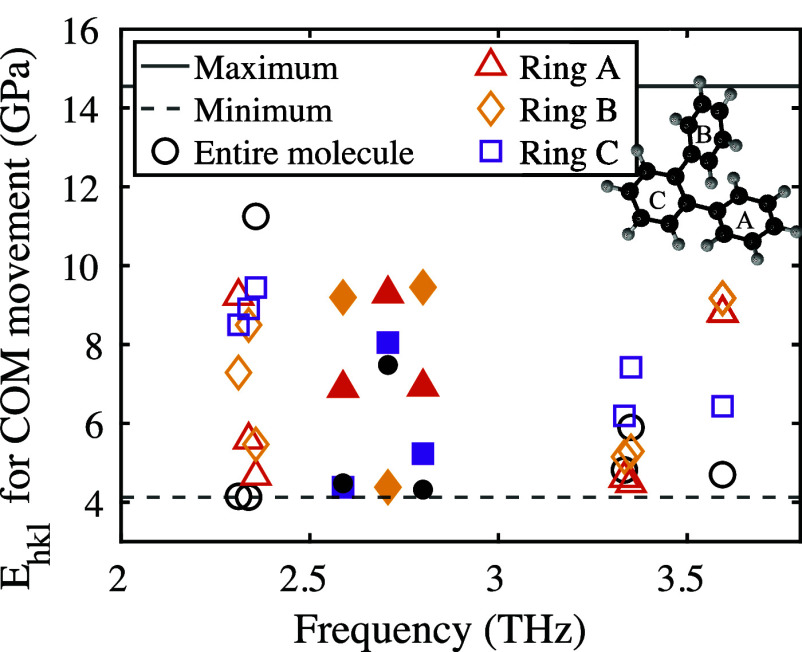
Young’s modulus in direction of center of mass movement for single rings and entire molecules for selected modes (IR-active: unfilled symbols, Raman-active: filled symbols).

This analysis provides some interesting results which can be correlated with the mode decomposition discussed above. For example, the direction of the COM motion of the entire molecule of mode at 2.36 THz is more rigid than for the two side rings. This correlates with the mode decomposition: the character of this mode is 89% intramolecular, while rotations and translations of the entire molecule amount to only 11%. The influence of intermolecular motions is higher for the modes at 2.31 THz (57% intermolecular) and 2.34 THz (45% intermolecular), and correspondingly the COM motion of the whole molecule occurs along a more elastic direction than that of the single rings.

To obtain physically meaningful normal mode contributions to the elastic tensor, we further decomposed the elastic stiffness constants within the Born–Oppenheimer approximation into a purely electronic and a nuclear “internal-strain” term.^74,75^ The nuclear term is evaluated by computing the internal-strain and Hessian tensors and partitioned into vibrational normal mode contributions.^76−79^ Details about this can be found in the Supporting Information.This analysis allows us to identify the lattice vibrations with the highest contribution to the elastic moduli; they are highlighted in Figure 4, with animations of the modes available in the Supporting Information. From this, we conclude that the electronic contribution to the elastic constants is largely positive, and the nuclear contribution is mostly negative. Major contributions to the elastic moduli in the terahertz regime involve collective vibrations and dihedral angle changes. The calculated stiffness tensor C_*ij*_ connects stress and strain in the generalized form of Hooke’s law.^73^ We observed that all IR-active modes are pure shear modes, i.e. involve only one of the stiffness components C_44_, C_55_, or C_66_. In contrast, Raman-active modes (those which are not IR active) also involve off-diagonal elements of the C_*ij*_ tensor and are thus not pure shear modes. Comparison of the modes highlighted in Figure 4 with Figure 1 reveals that these modes have a high intramolecular character, i.e., that they represent *internal* shear motions.

**Figure 4:**
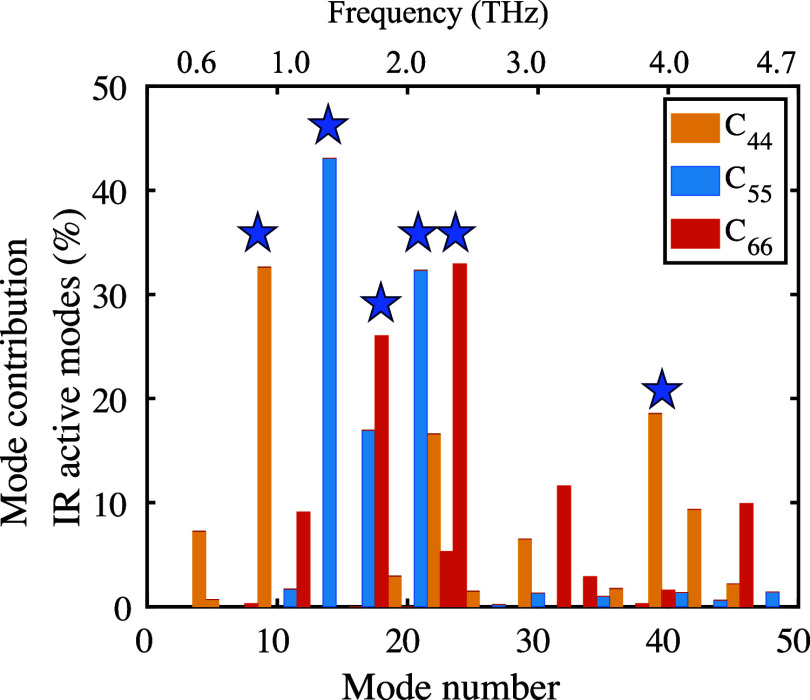
Harmonic normal mode contributions to the elastic moduli of OTP. The lattice vibrations with the largest contribution in the terahertz range are highlighted with stars.

The experiments and simulations discussed above all serve to highlight a few key points. There are a variety of internal modes in the terahertz range active in crystalline OTP even at very low temperatures. We furthermore observe active torsional motions, especially at higher temperatures. Unlike crystals, liquids and glasses do not possess either long-range translational nor orientational order. However, they still tend to form local structural order.^80−84^ This lowers the free energy locally and, in the case of entropically driven ordering, can lead to extended structures with orientational order.^80^ In such disordered solids, there is an abundance of low-frequency quasi-localized modes, which often lie in the terahertz range.^85,86^ Those modes as well as the elasticity of a disordered solid are a direct consequence of the shape and structure of the PES, which also determines glass-forming dynamics.

*Amorphous OTP*. We have studied the glass transition in OTP. In the disordered (glassy) state, the IR- and Raman-active modes described above are also active, but no longer coherent. Instead, they contribute to an increase of the absorption coefficient with temperature in the terahertz range. To study the behavior of disordered OTP, melted OTP was filled into a sample cell (thickness 600 μm) and quench cooled to 80 K within 30 minutes. The absence of spectral features in the terahertz spectrum confirmed that no crystallization occurred during cooling.^87^ The sample was then slowly heated and measurements were taken at temperature intervals of 10 K. The corresponding terahertz absorption spectra, essentially featureless below the glass transition, are shown in Figure S5. The onset of crystallization was observed at a temperature of approximately 250 K, indicated by the emergence of a spectral feature at 2.5 THz, and melting occurred at 328 K. Previous studies have found a glass transition temperature, *T*_g,α_, of 243 K, as well as a secondary glass transition temperature, *T*_g,β_, of 133 K.^10,11,72,88^ These temperatures are shown together with experimental data in Figure 5. We note that a *T*_g,β_ of 133 K is rather low; for most small organic molecular systems, it lies between 150 to 250 K.^89^ This is another indicator that intermolecular modes are active and already contribute to molecular mobility at very low temperatures. We do not observe any peaks emerging in the THz data at *T*_g,β_, which is consistent with the understanding that the accompanying increase in molecular mobility does not lead to the emergence of specific features but rather to an overall increase in the vibrational density of states.^90^ In our experiments (average heating rate approximately 1 K min^–1^), crystallization began within a few minutes of the system reaching a temperature above *T*_g,α_ (243 K). It was previously reported that crystal growth from nuclei can occur in OTP even below *T*_g,α_, at temperatures as low as 225 K, which they argue stems from molecular rearrangement by the β-relaxation rather than the α-process, as the temperature is below the glass transition temperature.^91−93^ This means that the mix of internal and external modes active below *T*_g,α_ is sufficient to drive crystal growth in OTP.

**Figure 5:**
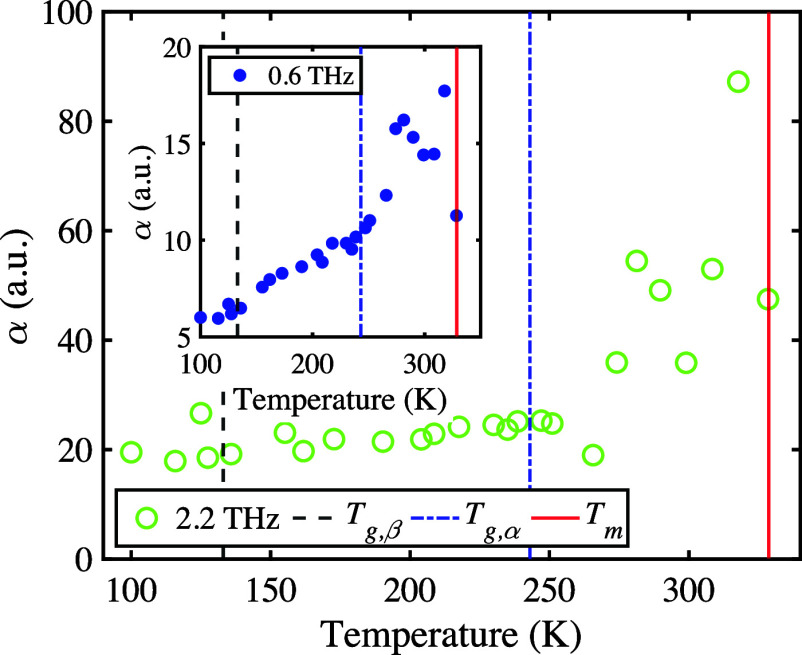
Absorption coefficient at selected frequencies for different temperatures. Previously reported glass transition and melting temperatures have been marked with vertical lines.^88^

*Conclusions.* We report a detailed study of the internal modes of OTP in both the crystalline and glassy states, using terahertz time-domain spectroscopy and low-frequency Raman spectroscopy. The experimental spectra taken at 300 and 80 K show a number of IR- and Raman-active resonances in the crystal, which could be assigned based on numerical simulations. We performed DFT simulations of the crystalline state at 0 K, and used AIMD simulations to evaluate the system at higher temperatures. The positions of experimentally determined modes were reproduced well in all cases in the numerical simulations. We show that the majority of modes in the terahertz range contain mixed inter- and intramolecular displacements, and modes with an intramolecular character dominate above 3 THz. Torsions, i.e., dihedral angle changes between OTP’s central and side rings, are active at room temperature and also are dominant at frequencies above 3 THz. Decomposition of the calculated elastic tensor revealed that all IR-active modes are pure shear modes. Crucially, these modes are also present in disordered OTP where they contribute to inter- and intramolecular mobility even at cryogenic temperatures.

These results call into question the generally accepted assumption that OTP can be well approximated as a completely rigid molecule which is linked to its neighbors only by nondirectional van der Waals interactions (or, at least, that all of its internal degrees of freedom can be neglected in treatments of glass formation, as in mode coupling theory). Evidently, MCT has been very successful in describing many aspects of glass formation dynamics. Yet, one of its key shortcomings is its well-known inaccuracy in predicting transition temperatures.^4^ Incorporating internal molecular degrees of freedom may be important in order to improve MCT. We show that by mapping out the potential energy surface with far-infrared spectroscopies and simulations, we can gain insight into the elasticity and dynamics of the model system OTP.

## Supporting Information Available

Figure S1: Comparison of extinction coefficient at 300 K, 80 K, and calculated with DFT
Figure S2: Comparison of Raman shift at 175 and 300 K
Figure S3: Spectra calculated from AIMD trajectories at different temperatures
Figure S4: Visualization of the anisotropy of Young’s modulus of OTP
Figure S5: Absorption spectra of glassy OTP during heating
Additional information on methods (PDF)
gifs for high-intensity IR modes
gifs for high-intensity Raman modes
gifs for low-elastic moduli modes
.xyz files for all animations(ZIP)
