# Supplementary material for: Is Ortho-Terphenyl a Rigid Glass Former?
Source: J Phys Chem Lett. 2024 Jul 1;15(27):7020–7. doi: 10.1021/acs.jpclett.4c01217 (PMC11247491; doi:10.1021/acs.jpclett.4c01217)
Supplement: Supplementary file 1 — jz4c01217_si_001.pdf [file jz4c01217_si_001.pdf]

Is Ortho-Terphenyl a Rigid Glass Former?

Johanna Kölbel,[1,2] Michael T. Ruggiero,[3] Shachar Keren,[4] Nimrod Benshalom,[4] Omer Yaffe,[4] J. Axel Zeitler,[2] and Daniel Mittleman[1]

[1] *School of Engineering, Brown University, Providence, RI, 02912 USA*
[2] *Department of Chemical Engineering and Biotechnology, University of Cambridge, Cambridge, CB3 0AS UK*
[3] *Department of Chemistry, University of Rochester, Rochester, New York, 14627 USA*
[4] *Department of Chemical and Biological Physics, Weizmann Institute of Science, Rehovot, Israel*
(Dated: June 21, 2024)

- Comparison of extinction coefficient at 300 K, 80 K, and calculated with DFT.
- Comparison of Raman shift at 175 K and 300 K.
- Spectra calculated from AIMD trajectories at different temperatures.
- Visualization of the anisotropy of Young's modulus of OTP.
- Absorption spectra of glassy OTP during heating.
- Methods
- gifs for high-intensity IR modes
- gifs for high-intensity Raman modes
- gifs for low-elastic moduli modes
- .xyz files for all animations

SUPPLEMENTARY FIGURES

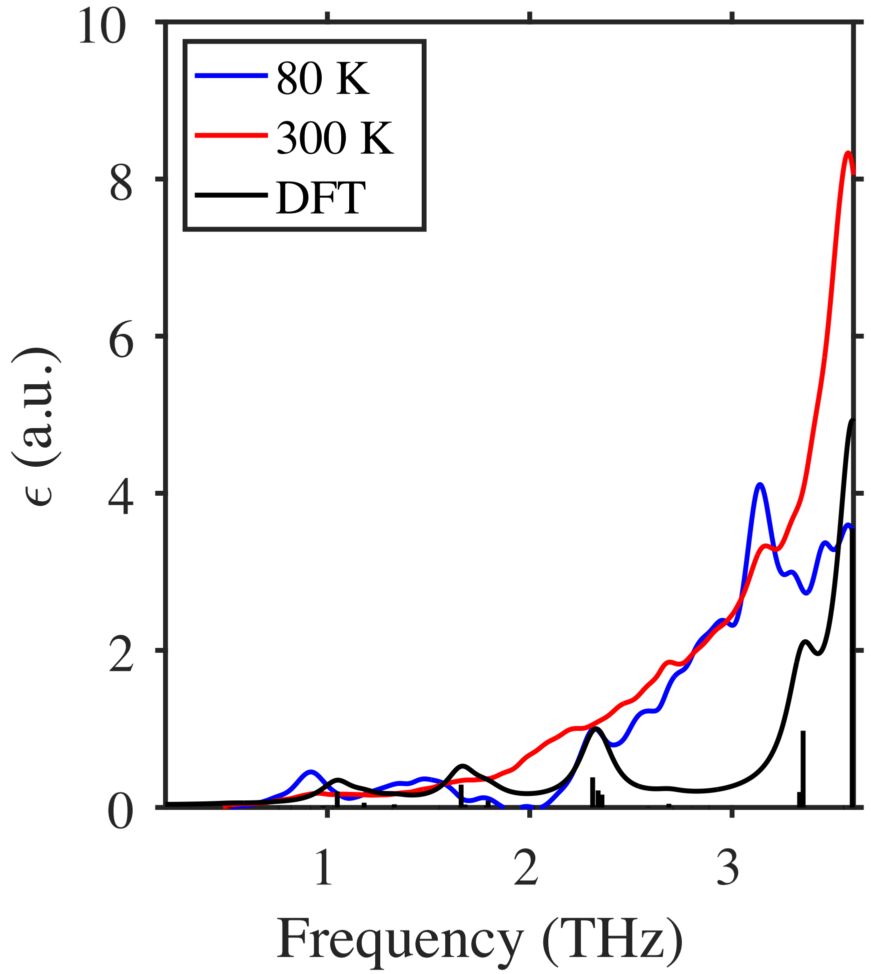

Figure S1. Comparison of extinction coefficient at 300 K (red), 80 K (blue), and calculated with DFT (black). Intensities have been scaled to the peak at 2.3 THz. The calculated intensities (black sticks) have been convolved with Lorentzian line shapes using the empirical FWHM value from the experimental spectrum.

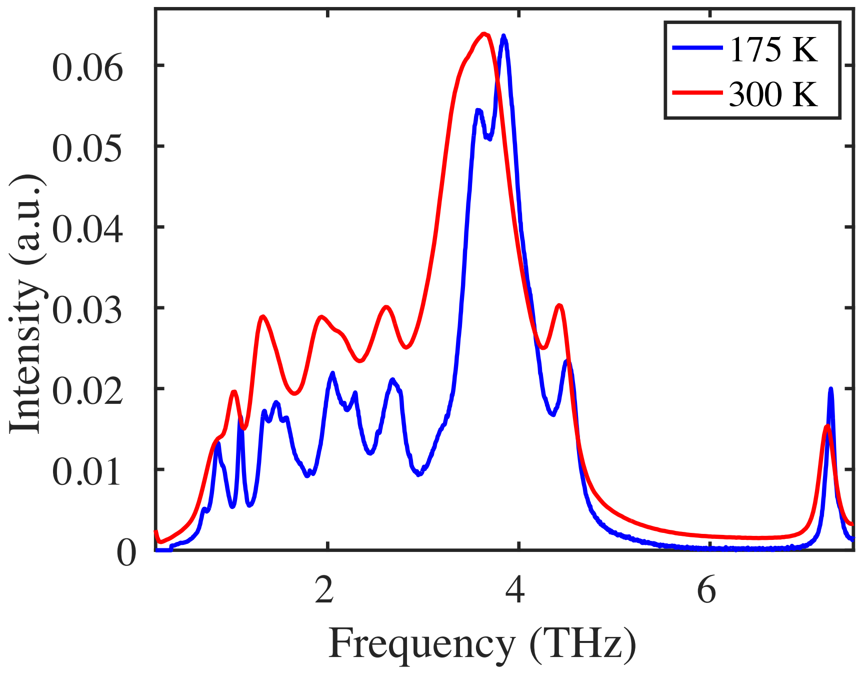

Figure S2. Comparison of Raman shift at 175 K (blue) and 300 K (red).

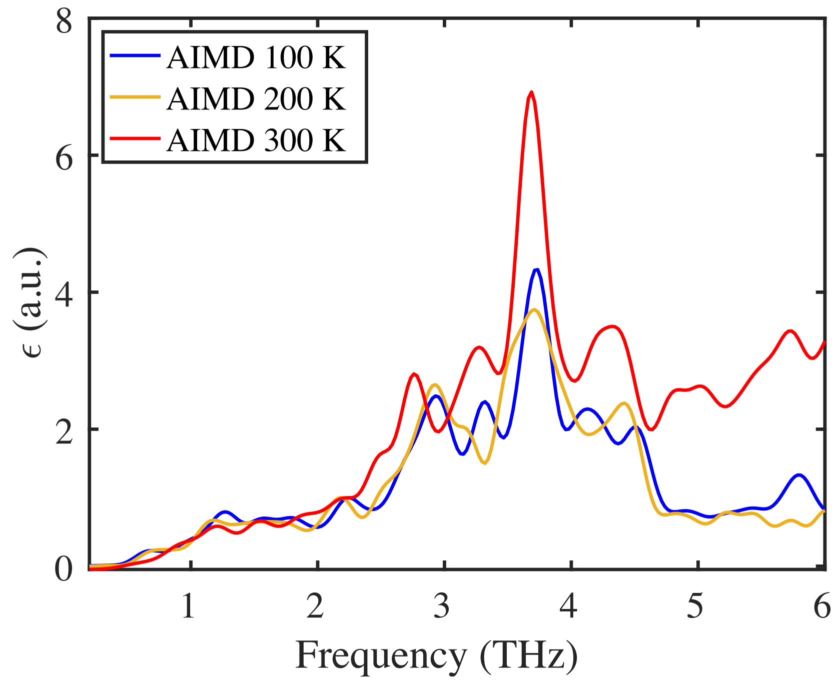

Figure S3. Spectra calculated from AIMD trajectories at different temperatures.

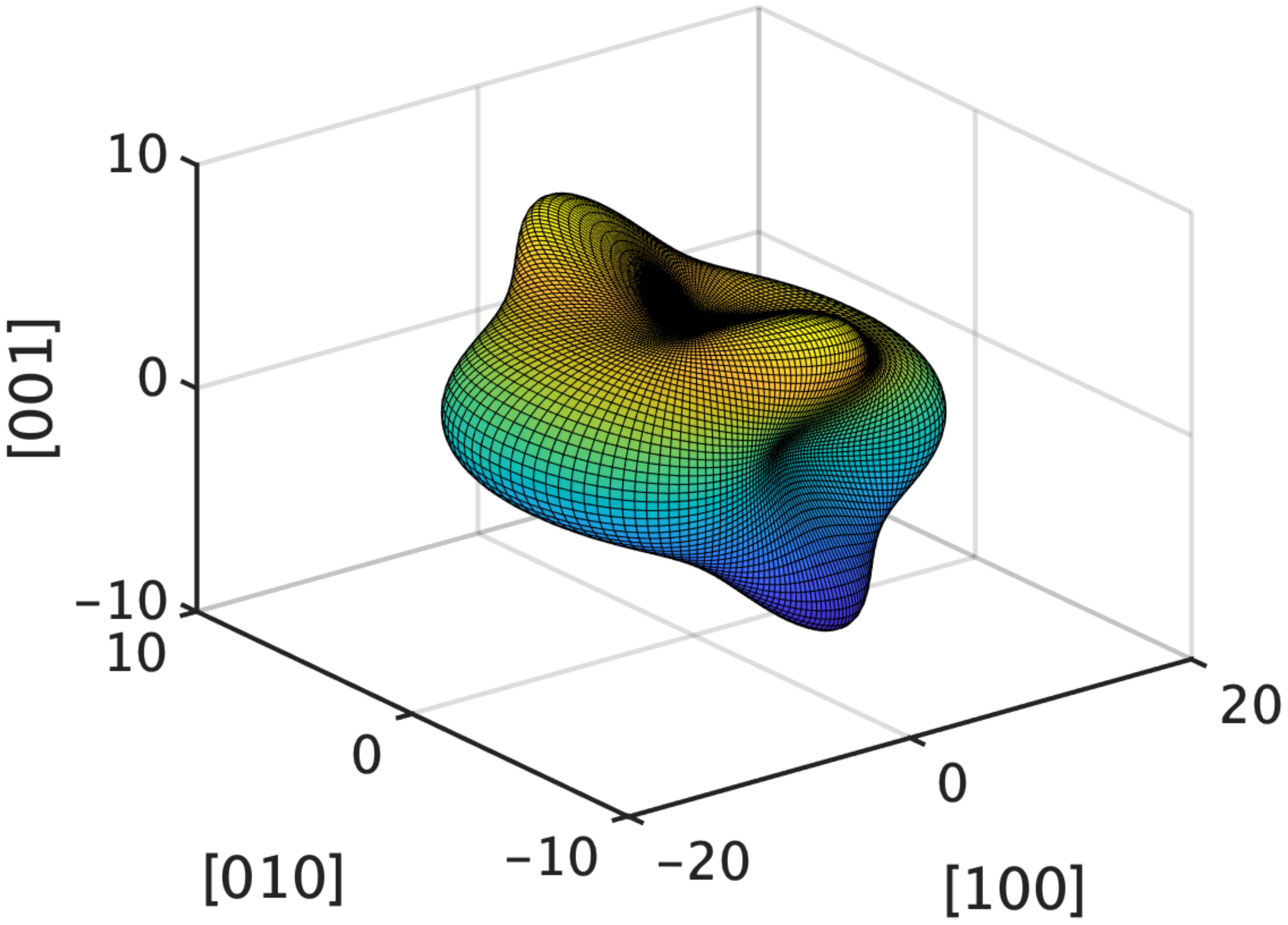

Figure S4. Visualization of the anisotropy of Young's modulus of OTP.

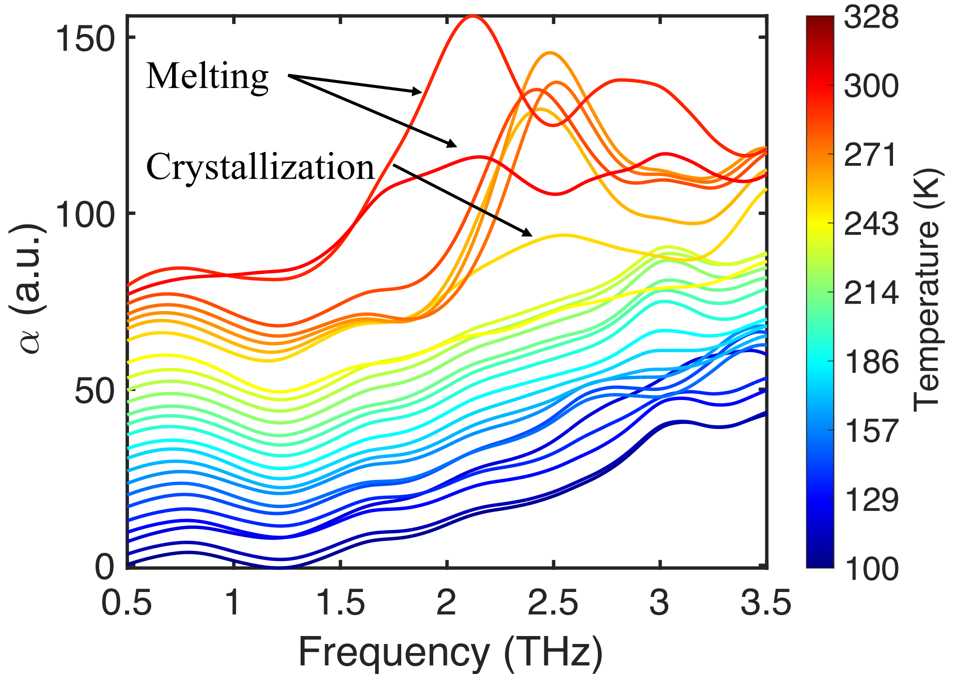

Figure S5. Absorption spectra of glassy OTP during heating. Subsequent spectra are offset for better visibility and the onset of crystallization and melting is marked.

METHODS

Terahertz Time-Domain Spectroscopy

Pure OTP was acquired from TCI America and ground to powder using a mortar and pestle for measurements of its crystalline form. The powder was subsequently placed in a hydraulic press and made into a 1 mm thick pellet with a diameter of 10 mm. The pellet was placed into a cryostat and measured under vacuum with a terahertz time domain spectrometer, with a usable bandwidth of 0.3 THz to 5 THz at 80 K.

For measurements in the glassy state, melted OTP was filled into a liquid cell (thickness 600 µm) and quench cooled to 80 K within 30 minutes. The absence of spectral features in the spectrum, as measured with the same THz spectrometer, confirmed that no crystallization occurred during cooling. The sample was then slowly heated and measurements were taken at temperature intervals of 10 K until melting was observed.

Raman Spectroscopy

Raman spectra were taken in a home-built system using back-scattering measurement geometry, with a 785 nm CW diode laser (Toptica Inc., USA) with approximately 20 mW incident intensity. The beam was linearly polarized (Thorlabs, USA), with unpolarized spectra given by averaging over all incident polarizations. The beam was focused on the samples with 50X objectives (Zeiss, USA). Rayleigh scattering was filtered by notch filters (Ondax Inc., USA) with the beam finally focused into a 1 m spectrometer (FHR 1000, Horiba) with a 1800 g/mm grating for 175 K, and 600 g/mm grating for room temperature measurements, and detected by a Synapse CCD.

Density Functional Theory

The CRYSTAL17 software package was used together with the Perdew-Burke-Ernzerhof (PBE) density functional [1] and Ahlrich-VTZP basis set [2] to perform the static-DFT calculations. D3-dispersion correction [3] [4] was used to correct for the London dispersion force. Periodic boundary conditions ensure that the translational symmetry of the crystal is preserved and were used in all simulations [5].

Initial atomic positions and lattice parameters were taken from published crystallographic data (CSD) [6]. The reciprocal space sampling was tested and a 6 x 6 x 6 Monkhorst-Pack grid [7] was chosen to evaluate the reciprocal space during the simulation. The atomic positions were then optimized with no constraints other than the space-group symmetry of OTP and with an energy convergence criterion of 10^{-8} hartree. The cell lengths of the optimized geometry deviated by 0.07 % (a), 3.77 % (b), and 2.88 % (c) from the experimental structure, respectively.

The root mean square deviation (RMSD) of the bond lengths was 0.01 Å. Typical bond lengths in OTP are approximately 1.1 Å (H-C) to 1.4 Å (C-C), so the deviation in bond lengths was only about 1 % to 1.5 %. The angles between the atoms also changed, with a RMSD of about 0.49°, and the RMSD of the dihedrals was 3.39°, resulting from torsion of the phenyl groups.

A vibrational analysis was performed by calculation of the second derivative of the potential energy surface via a numerical finite difference scheme [8] [9], using a three-point central difference method (two displacements per atom per cartesian axis). Vibrational normal mode eigenvectors and eigenvalues were calculated within the harmonic approximation through diagonalization of the mass-weighted Hessian (force constant) matrix and IR intensities were calculated via the Berry Phase method.[10] The energy convergence limit was set to 10^{-10} hartree.

The Raman specta were calculated analytically using the coupled-perturbed Kohn-Sham method in CRYSTAL23.[11–13] The Raman spectra presented were generated with Lorentzian functions, with a full-width at half-maximum of 3.0 cm^{-1}. The spectra were generated using the experimental laser wavelength (785 nm) and the Stokes to anti-Stokes ratios were determined using the temperature of the experiment (175 K).

The elastic tensor was calculated numerically utilizing a fully-automatic procedure in CRYSTAL23[14–16]. The elastic constants (C_{ij} were partitioned into contributions from individual vibrational modes using a previously described procedure. This enabled correlating the various vibrational normal modes with their contribution to the elastic tensor elements.[14, 17–19]

AIMD

The optimized geometry calculated by DFT was used as the initial input for atoms and lattice parameters. A supercell containing 256 atoms was defined. For improved comparability with DFT calculations, the PBE functional [1] was combined with D3 dispersion correction [4], the DZVP basis set [20] and Goedecker-Teter-Hutter (GTH) pseudopotentials for core electrons [21]. Calculations were performed utilizing the CP2K software package [22, 23].

After being optimized at 0 K, the structure was allowed to relax and equilibrate at 100 K, 200 K, and 300 K, for 10 ps to 12 ps respectively, within an isothermal-isobaric (NPT) ensemble with a time step of 1 fs and a time constant of 50. [24]

Cell lengths were monitored and after a stabilization period, average cell lengths were extracted for each temperature, and further simulations were performed within the canonical (NVT) ensemble. The duration of each time step was lowered to 0.5 fs to increase accuracy and a Wannier localization was performed every 2.5 fs to compute the molecular dipole moments. Post-processing of the simulated trajectories including the vibrational analysis at 100 K, 200 K, and 300 K was performed with TRAVIS [24, 25].

Calculation of Young's modulus for arbitrary strain directions

Hooke's law states that the application of a general homogenous stress σ_{ij} to a crystal results in a homogenous strain ϵ_{ij}:

$$\epsilon_{ij} = s_{ijkl}\sigma_{kl} \tag{1}$$

$$\sigma_{ij} = c_{ijkl}\epsilon_{kl} \tag{2}$$

with c_{ijkl} stiffness constants (Young's modulus) and s_{ijkl} compliances (Einstein summation convention applies). Depending on the crystal space group, several of those components are 0 or not independent of each other. The DFT simulations have provided us with the values of c_{ijkl} and s_{ijkl} (CRYSTAL keyword ELASTCON). As given by Nye [26], the reciprocal of Young's modulus in the direction of the unit vector $\vec{l_i} = \begin{pmatrix} l_1 \\ l_2 \\ l_3 \end{pmatrix}$ in an orthorhombic crystal system (such as OTP) is:

$$\frac{1}{E_{\text{direction}}} = l_1^4 s_{11} + 2l_1^2 l_2^2 s_{12} + 2l_1^2 l_3^2 s_{13} + l_2^4 s_{22} + 2l_2^2 l_3^2 s_{23} + l_3^4 s_{33} + l_2^2 l_3^2 s_{44} + l_1^2 l_3^2 s_{55} + l_1^2 l_2^2 s_{66} \tag{3}$$

Where the s_{ij} are the Voigt notation of the s_{ijkl}.[26] We therefore first calculated the direction corresponding to the the center-of-mass movement for a certain normal mode from the optimized geometry from our DFT output (by comparing the equilibrium geometry and the geometry after the normal mode displacement, CRYSTAL keyword FREQCALC). We then calculated Young's modulus for that particular strain direction according to Eqn. 3, as also visualized in Fig. S4 for all possible directions.

Decomposition of elastic stiffness constants

As Dal Corso et al. described in [27], in the Born-Oppenheimer approximation the macroscopic piezoelectric tensor results from two distinct contributions: the electronic contribution, which is evaluated at vanishing microscopic strain, and the nuclear contribution, which is due to the relative displacement of sublattices (in the example of ZnO), and is equivalent to a zone-center optic phonon. This distinction between the electronic and nuclear contribution is inherently applicable to a much wider range of materials within the Born-Oppenheimer approximation. Experimentally, there is no easy way to distinguish between the terms, but numerous simulations have calculated them and validated this as a well-established approach. [17, 28–33] The evaluation of the nuclear-relaxation term due to the rearrangement of atomic positions upon strain is usually the more computationally expensive of the two.

Within CRYSTAL23 [34, 35], the elements of the force-response internal-strain tensor are second-energy derivatives with respect to an atomic displacement and to a lattice distortion:

$$\Delta_{ai,v} = \frac{\partial E}{\partial u_{ai}\partial \eta_v}\Big|_{\epsilon} \tag{4}$$

where u_{ai} are Cartesian components of the displacement vector u_a of atom a (i=x, y, z).

A displacement-response internal-strain tensor Γ, which describes first-order atomic displacements as induced by a first-order strain, can be defined as:

$$\Gamma_{ai,v} = -\frac{\partial u_{ai}}{\partial \eta_v}\Big|_{\epsilon} = \sum_{bj} (H^{-1})_{ai,bj} \Delta_{bj,v} \tag{5}$$

where H is the interatomic force-constant Hessian matrix of energy second derivatives with respect to pairs of periodicity-preserving atomic displacements, which can also be calculated within CRYSTAL (keywords NUCHESS and HESSNUM2 [17, 18]).

The nuclear-relaxation contribution to elastic constants can then be expressed in terms of the internal-strain tensor Δ (or Γ):

$$C^{\text{nuc}}_{vw} = -\frac{1}{V_0} \sum \Delta_{ai,v} \Gamma_{ai,w} \tag{6}$$

The elements $\Delta_{ai,v}$ of the force-response internal-strain tensor are computed as finite differences of analytical lattice gradients with respect to atomic Cartesian displacements, by means of a generalized "Pulay's force method" originally proposed for interatomic force constants. [18, 36]

[1] J. P. Perdew, K. Burke, and M. Ernzerhof, "Generalized gradient approximation made simple," *Phys. Rev. Lett.*, vol. 77, pp. 3865–3868, 1996.

[2] A. Schaefer, H. Horn, and R. Ahlrichs, "Fully optimized contracted Gaussian basis sets for atoms Li to Kr," *J. Chem. Phys.*, vol. 97, pp. 2571–2577, 1992.

[3] R. J. Maurer, V. G. Ruiz, and A. Tkatchenko, "Many-body dispersion effects in the binding of adsorbates on metal surfaces," *J. Chem. Phys.*, vol. 143, p. 102808, 2015.

[4] S. Grimme, S. Ehrlich, and L. Goerigk, "Effect of the damping function in dispersion corrected density functional theory," *J. Comput. Chem.*, vol. 32, pp. 1456–1465, 2011.

[5] R. Dovesi, A. Erba, R. Orlando, C. M. Zicovich-Wilson, B. Civalleri, L. Maschio, M. Rérat, S. Casassa, J. Baima, S. Salustro, *et al.*, "Quantum-mechanical condensed matter simulations with crystal," *Wiley Interdiscip. Rev. Comput. Mol. Sci.*, vol. 8, no. 4, p. e1360, 2018.

[6] S. Aikawa, Y. Maruyama, Y. Ihashi, and Y. Sasada, "1,2-diphenylbenzene (o-terphenyl)," *Acta Cryst.*, vol. 34(9), pp. 2901–2904, 1978.

[7] H. J. Monkhorst and J. D. Pack, "Special points for Brillouin-zone integrations," *Phys. Rev. B*, vol. 13, pp. 5188–5192, 1976.

[8] C. M. Zicovich-Wilson, F. Pascale, C. Roetti, V. R. Saunders, R. Orlando, and R. Dovesi, "Calculation of the vibration frequencies of alpha-quartz: The effect of Hamiltonian and basis set," *J. Comput. Chem.*, vol. 25, pp. 1873–1881, 2004.

[9] F. Pascale, C. M. Zicovich-Wilson, F. Lopez-Gejo, B. Civalleri, R. Orlando, and R. Dovesi, "The calculation of the vibrational frequencies of crystalline compounds and its implementation in the CRYSTAL code," *J. Comput. Chem.*, vol. 25, pp. 888–897, 2004.

[10] Y. Noel, C. Zicovich-Wilson, B. Civalleri, P. Arco, and R. Dovesi, "Polarization properties of ZnO and BeO: An ab initio study through the Berry phase and Wannier functions approaches," *Phys. Rev. B: Condens. Matter Mater. Phys.*, vol. 65, pp. 1–9, 2001.

[11] L. Maschio, B. Kirtman, M. Rérat, R. Orlando, and R. Dovesi, "Ab initio analytical Raman intensities for periodic systems through a coupled perturbed Hartree-Fock/Kohn-Sham method in an atomic orbital basis. I. Theory," *J. Chem. Phys*, vol. 139, no. 16, 2013.

[12] L. Maschio, B. Kirtman, M. Rérat, R. Orlando, and R. Dovesi, "Ab initio analytical Raman intensities for periodic systems through a coupled perturbed Hartree-Fock/Kohn-Sham method in an atomic orbital basis. II. Validation and comparison with experiments," *J. Chem. Phys*, vol. 139, p. 164102, 10 2013.

[13] L. Maschio, B. Kirtman, M. Rérat, R. Orlando, and R. Dovesi, "Comment on "Ab initio analytical infrared intensities for periodic systems through a coupled perturbed Hartree-Fock/Kohn-Sham method"[J. Chem. Phys. 137, 204113 (2012)]," *J. Chem. Phys*, vol. 139, no. 16, 2013.

[14] J. Maul, M. R. Ryder, M. T. Ruggiero, and A. Erba, "Pressure-driven mechanical anisotropy and destabilization in zeolitic imidazolate frameworks," *Phys. Rev. B*, vol. 99, no. 1, p. 014102, 2019.

[15] W. Perger, J. Criswell, B. Civalleri, and R. Dovesi, "Ab-initio calculation of elastic constants of crystalline systems with the crystal code," *Comput. Phys. Commun.*, vol. 180, no. 10, pp. 1753–1759, 2009.

[16] A. Erba, A. Mahmoud, R. Orlando, and R. Dovesi, "Elastic properties of six silicate garnet end members from accurate ab initio simulations," *Phys. Chem. Miner.*, vol. 41, pp. 151–160, 2014.

[17] A. Erba, D. Caglioti, C. M. Zicovich-Wilson, and R. Dovesi, "Nuclear-relaxed elastic and piezoelectric constants of materials: Computational aspects of two quantum-mechanical approaches," *J. Comput. Chem.*, vol. 38, no. 5, pp. 257–264, 2017.

[18] A. Erba, "The internal-strain tensor of crystals for nuclear-relaxed elastic and piezoelectric constants: on the full exploitation of its symmetry features," *Phys. Chem. Chem. Phys.*, vol. 18, no. 20, pp. 13984–13992, 2016.
[19] X. Wu, D. Vanderbilt, and D. Hamann, "Systematic treatment of displacements, strains, and electric fields in density-functional perturbation theory," *Phys. Rev. B*, vol. 72, no. 3, p. 035105, 2005.
[20] N. Godbout, D. R. Salahub, J. Andzelm, and E. Wimmer, "Optimization of Gaussian-type basis sets for local spin density functional calculations. Part I. Boron through neon, optimization technique and validation," *Can. J. Chem.*, vol. 70, no. 2, pp. 560–571, 1992.
[21] S. Goedecker, M. Teter, and J. Hutter, "Separable dual space Gaussian pseudo-potentials," *Phys. Rev. B: Condens. Matter Mater. Phys.*, vol. 54, pp. 1703–1710, 1996.
[22] J. Hutter, M. Iannuzzi, F. Schiffmann, and J. Vandevondele, "CP2K: atomistic simulations of condensed matter systems," *Wiley Interdiscip. Rev. Comput. Mol. Sci.*, vol. 4, pp. 15–25, 2014.
[23] J. Vandevondele, M. Krack, F. Mohamed, M. Parrinello, T. Chassaing, and J. Hutter, "Quickstep: Fast and accurate density functional calculations using a mixed Gaussian and plane waves approach," *Comput. Phys. Commun.*, vol. 167, pp. 103–128, 2005.
[24] M. Thomas, M. Brehm, R. Fligg, P. Voehringer, and B. Kirchner, "Computing vibrational spectra from ab initio molecular dynamics," *Phys. Chem. Chem. Phys.*, vol. 15, pp. 6608–6622, 2013.
[25] M. Brehm and B. Kirchner, "TRAVIS - a free analyzer and visualizer for Monte Carlo and molecular dynamics trajectories," *J. Chem. Inf. Model.*, vol. 51, pp. 2007–2023, 2011.
[26] J. F. Nye, *Physical properties of crystals: their representation by tensors and matrices*. Oxford university press, 1985.
[27] A. Dal Corso, M. Posternak, R. Resta, and A. Baldereschi, "Ab initio study of piezoelectricity and spontaneous polarization in ZnO," *Phys. Rev. B*, vol. 50, no. 15, p. 10715, 1994.
[28] S. de Gironcoli, S. Baroni, and R. Resta, "Piezoelectric properties of iii-v semiconductors from first-principles linear-response theory," *Phys. Rev. Lett.*, vol. 62, no. 24, pp. 2853–2856, 1989.
[29] A. Erba, K. E. El-Kelany, M. Ferrero, I. Baraille, and M. Rérat, "Piezoelectricity of srtio 3: An ab initio description," *Phys. Rev. B*, vol. 88, no. 3, p. 035102, 2013.
[30] K. E. El-Kelany, A. Erba, P. Carbonnière, and M. Rérat, "Piezoelectric, elastic, structural and dielectric properties of the si1- xgexo2 solid solution: a theoretical study," *J. Condens. Matter Phys.*, vol. 26, no. 20, p. 205401, 2014.
[31] A. Erba, M. Ruggiero, T. Korter, and R. Dovesi, "Piezo-optic tensor of crystals from quantum-mechanical calculations," *J. Chem. Phys.*, vol. 143, no. 14, p. 144504, 2015.
[32] K. E. El-Kelany, P. Carbonnière, A. Erba, J.-M. Sotiropoulos, and M. Rerat, "Piezoelectricity of functionalized graphene: a quantum-mechanical rationalization," *J. Phys. Chem. C*, vol. 120, no. 14, pp. 7795–7803, 2016.
[33] A. Alsaad, I. Qattan, A. Ahmad, Q. M. Al-Bataineh, H. I. Al-Abed, Z. Albataineh, A. Telfah, and R. Sabirianov, "Theoretical and experimental overview of structural, dielectric, crystallographic, electronic, optical, and physical tensors of α-dipab and iodine-doped α-dipab molecular ferroelectric crystals," *J. Electron. Mater.*, vol. 49, pp. 7112–7132, 2020.
[34] R. Dovesi, V. Saunders, C. Roetti, R. Orlando, C. M. Zicovich-Wilson, F. Pascale, B. Civalleri, K. Doll, N. Harrison, I. Bush, P. D'Arco, M. Llunell, M. Causà, Y. Noeul, L. Maschio, A. Erba, M. Rerat, S. Casassa, B. Searle, and J. Desmarais, "Crystal23 user's manual," *University of Torino, Torino*, 2023.
[35] A. Erba, J. K. Desmarais, S. Casassa, B. Civalleri, L. Donà, I. J. Bush, B. Searle, L. Maschio, L. Edith-Daga, A. Cossard, *et al.*, "Crystal23: a program for computational solid state physics and chemistry," *J Chem. Theory Comput.*, vol. 19, no. 20, pp. 6891–6932, 2022.
[36] R. Dovesi, A. Erba, R. Orlando, C. M. Zicovich-Wilson, B. Civalleri, L. Maschio, M. Rérat, S. Casassa, J. Baima, S. Salustro, *et al.*, "Quantum-mechanical condensed matter simulations with crystal," *Wiley Interdiscip. Rev. Comput. Mol. Sci.*, vol. 8, no. 4, p. e1360, 2018.
