# Supplementary figures and images for: Is Ortho-Terphenyl a Rigid Glass Former?
Source: J Phys Chem Lett. 2024 Jul 1;15(27):7020–7. doi: 10.1021/acs.jpclett.4c01217 (PMC11247491; doi:10.1021/acs.jpclett.4c01217)

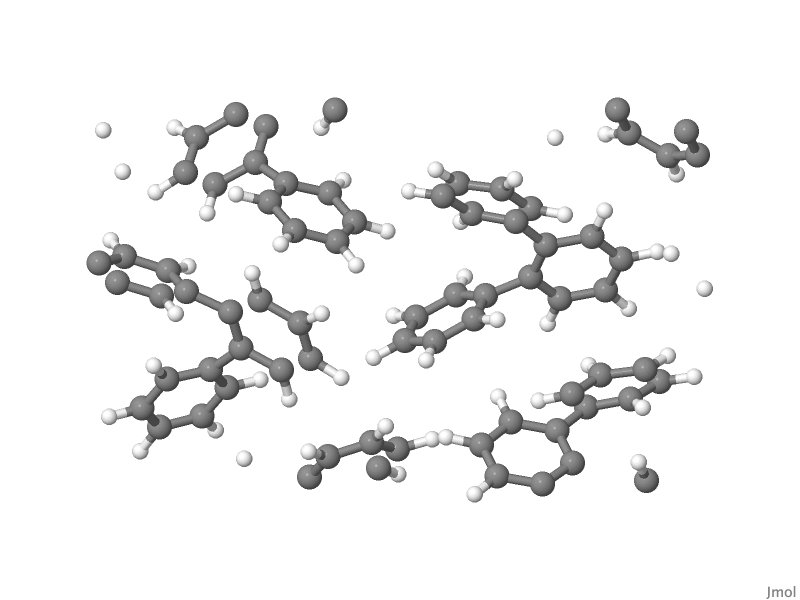

Supplement: Supplementary file 2 — jz4c01217_si_002.zip [file jz4c01217_si_002.zip › mode_animations/IR active/f32_new.gif]

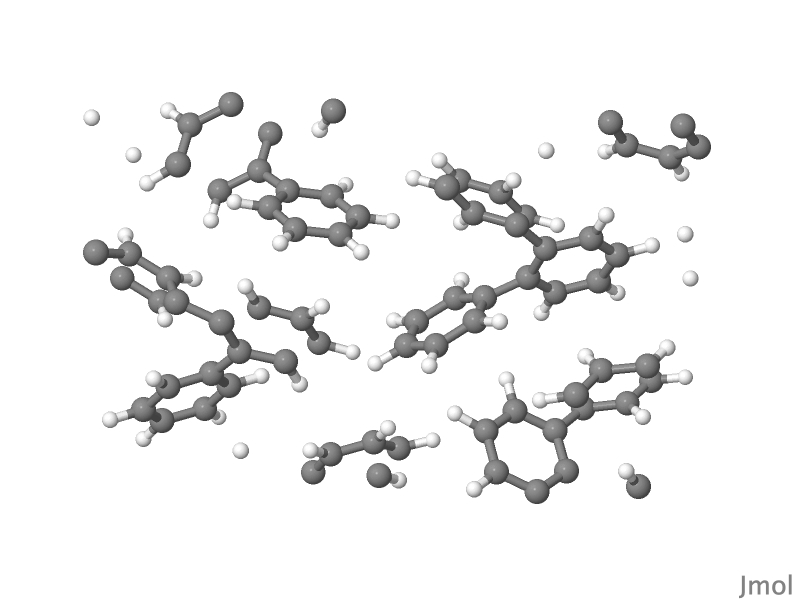

Supplement: Supplementary file 2 — jz4c01217_si_002.zip [file jz4c01217_si_002.zip › mode_animations/IR active/f29_new.gif]

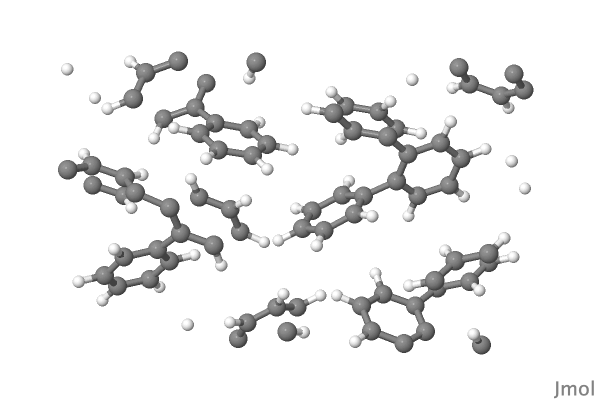

Supplement: Supplementary file 2 — jz4c01217_si_002.zip [file jz4c01217_si_002.zip › mode_animations/IR active/f21.gif]

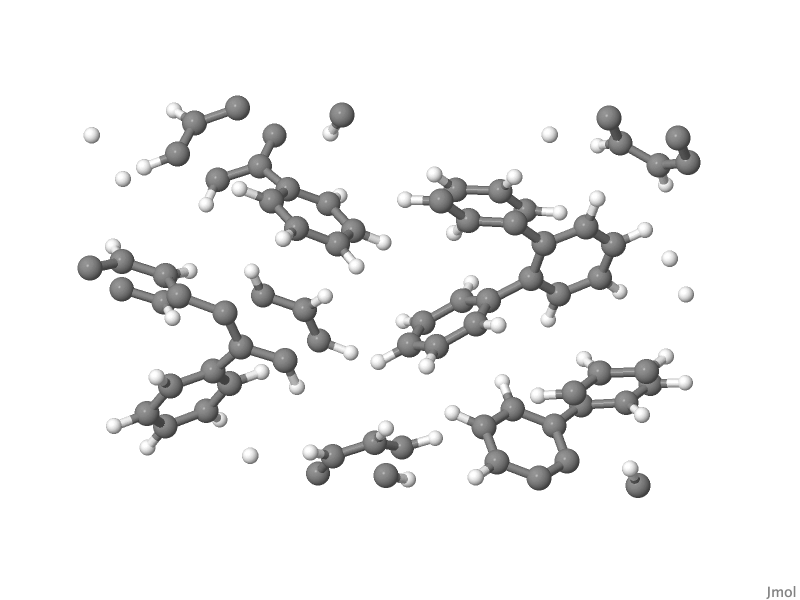

Supplement: Supplementary file 2 — jz4c01217_si_002.zip [file jz4c01217_si_002.zip › mode_animations/IR active/f23.gif]

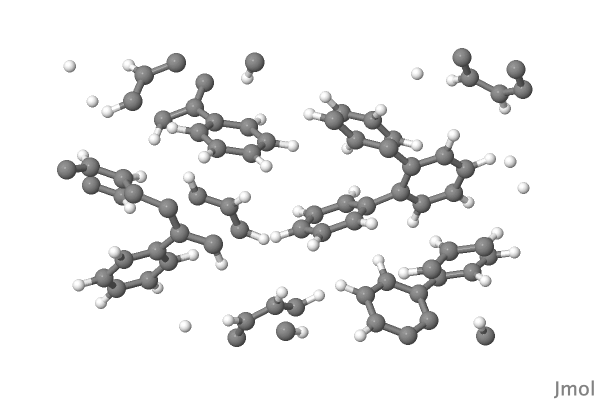

Supplement: Supplementary file 2 — jz4c01217_si_002.zip [file jz4c01217_si_002.zip › mode_animations/IR active/f22.gif]

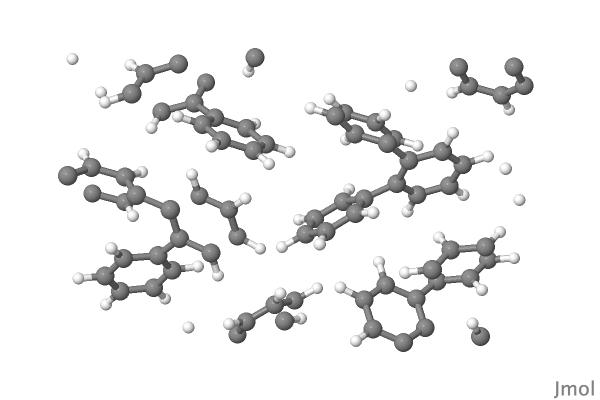

Supplement: Supplementary file 2 — jz4c01217_si_002.zip [file jz4c01217_si_002.zip › mode_animations/IR active/f30.gif]

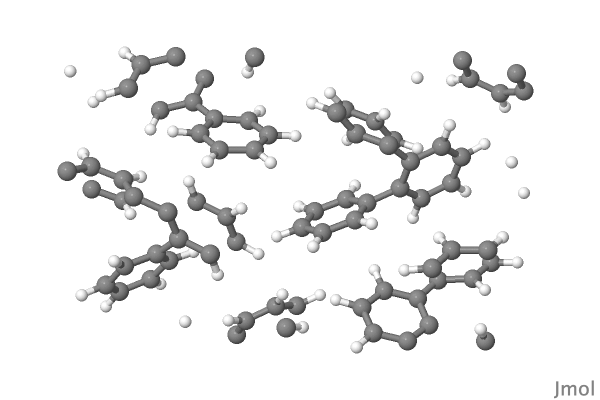

Supplement: Supplementary file 2 — jz4c01217_si_002.zip [file jz4c01217_si_002.zip › mode_animations/Raman active/f39.gif]

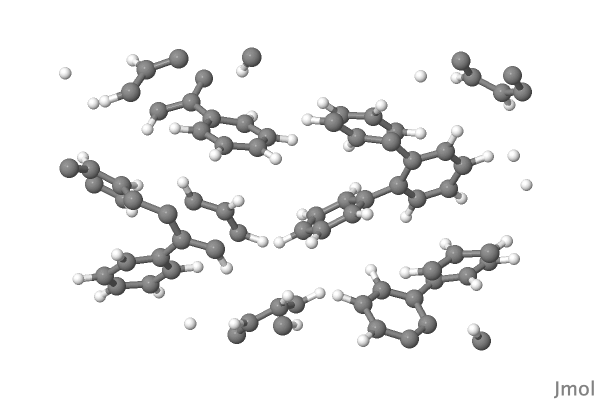

Supplement: Supplementary file 2 — jz4c01217_si_002.zip [file jz4c01217_si_002.zip › mode_animations/Raman active/f43.gif]

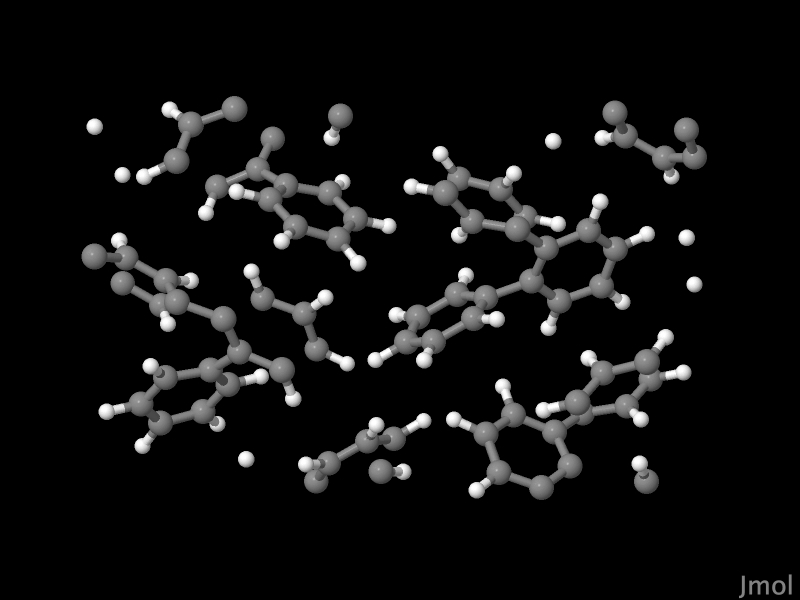

Supplement: Supplementary file 2 — jz4c01217_si_002.zip [file jz4c01217_si_002.zip › mode_animations/Raman active/f51.gif]

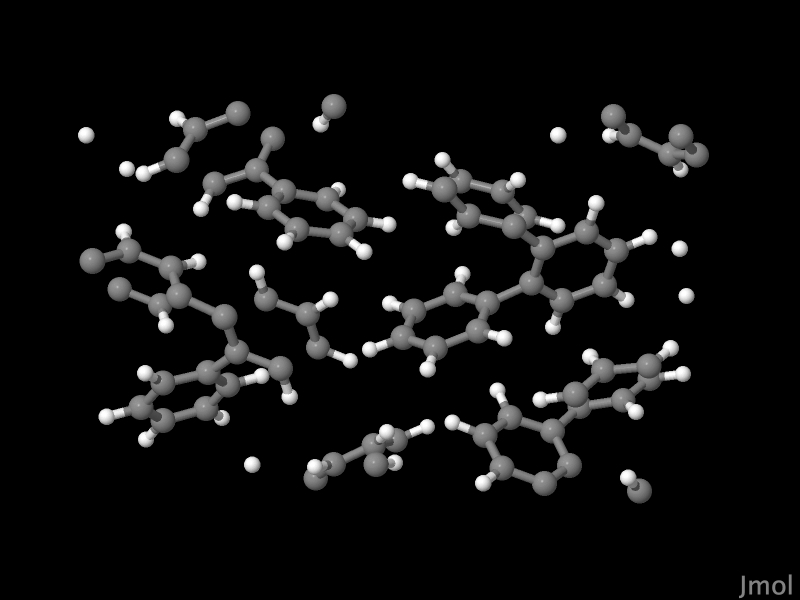

Supplement: Supplementary file 2 — jz4c01217_si_002.zip [file jz4c01217_si_002.zip › mode_animations/Raman active/f45.gif]

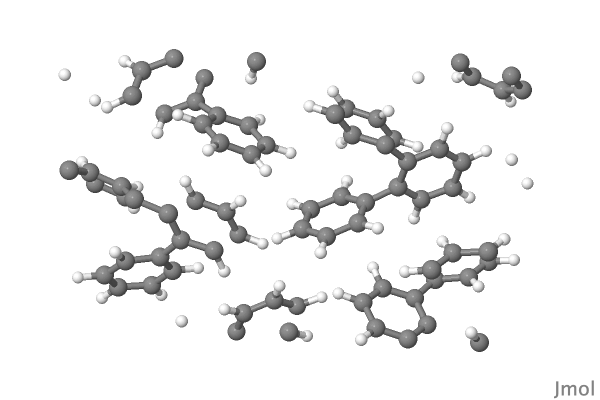

Supplement: Supplementary file 2 — jz4c01217_si_002.zip [file jz4c01217_si_002.zip › mode_animations/Raman active/f44.gif]

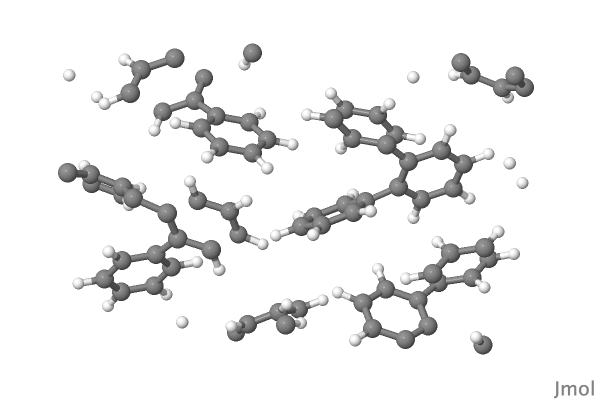

Supplement: Supplementary file 2 — jz4c01217_si_002.zip [file jz4c01217_si_002.zip › mode_animations/Raman active/f47.gif]

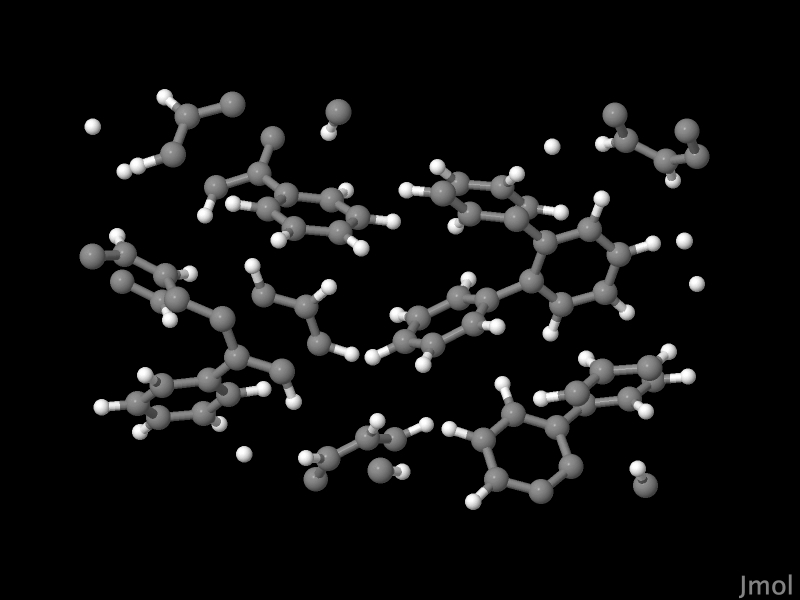

Supplement: Supplementary file 2 — jz4c01217_si_002.zip [file jz4c01217_si_002.zip › mode_animations/Raman active/f37.gif]

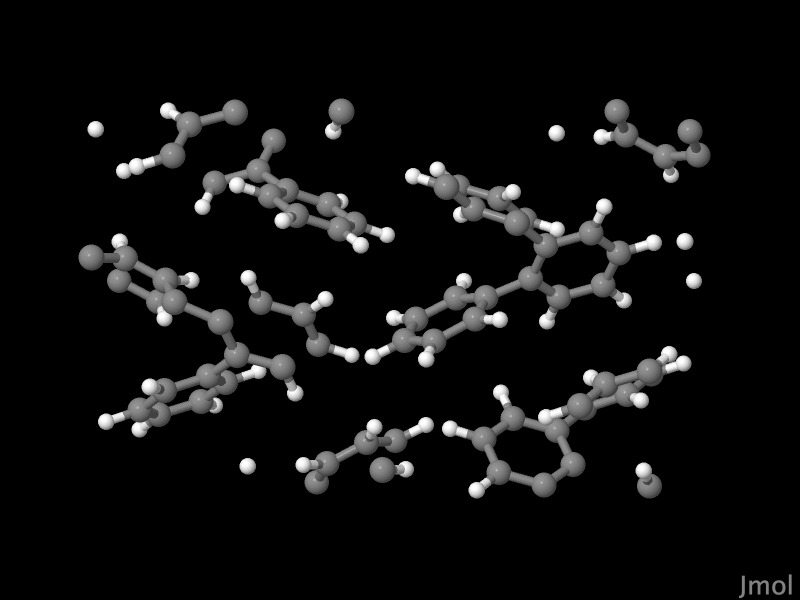

Supplement: Supplementary file 2 — jz4c01217_si_002.zip [file jz4c01217_si_002.zip › mode_animations/Raman active/f33.gif]

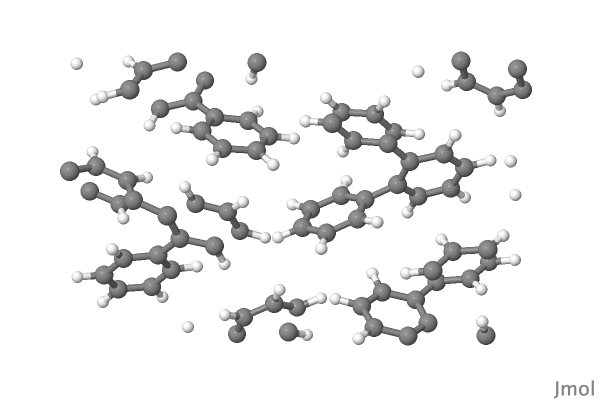

Supplement: Supplementary file 2 — jz4c01217_si_002.zip [file jz4c01217_si_002.zip › mode_animations/Raman active/f31.gif]

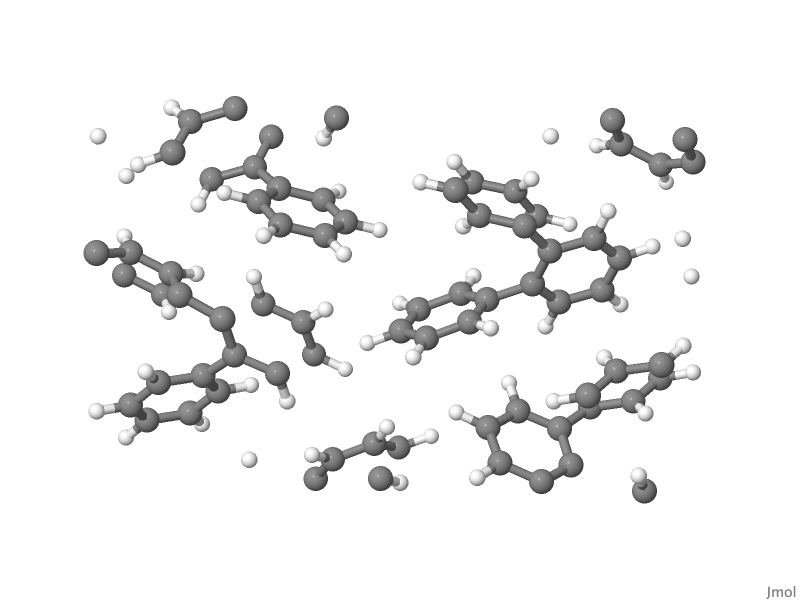

Supplement: Supplementary file 2 — jz4c01217_si_002.zip [file jz4c01217_si_002.zip › mode_animations/Mode contribution/f14.gif]

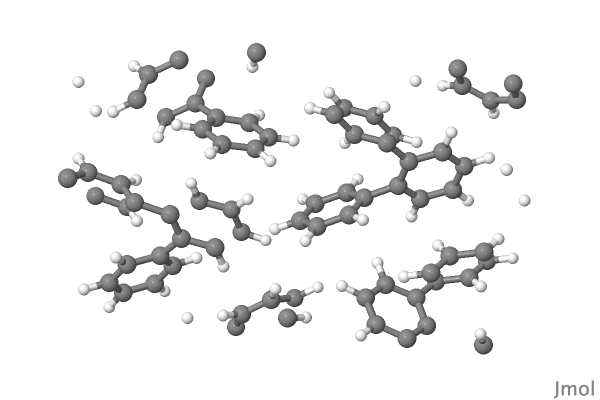

Supplement: Supplementary file 2 — jz4c01217_si_002.zip [file jz4c01217_si_002.zip › mode_animations/Mode contribution/f9.gif]

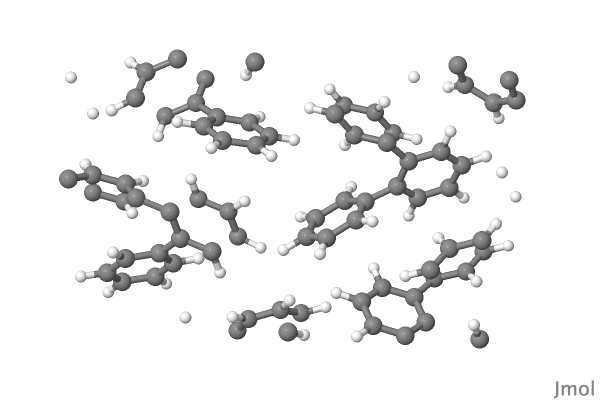

Supplement: Supplementary file 2 — jz4c01217_si_002.zip [file jz4c01217_si_002.zip › mode_animations/Mode contribution/f24.gif]

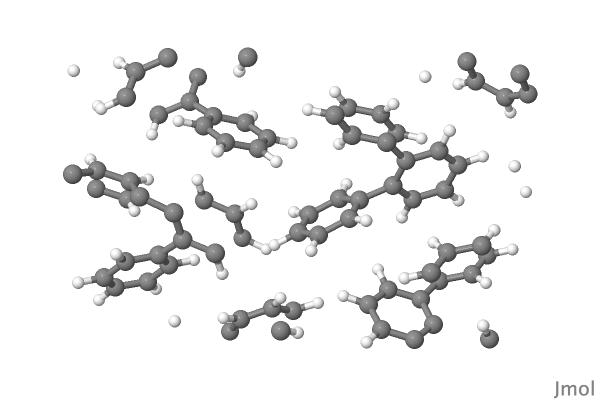

Supplement: Supplementary file 2 — jz4c01217_si_002.zip [file jz4c01217_si_002.zip › mode_animations/Mode contribution/f18.gif]
